# A host factor supports retrotransposition of the TRE5-A population in *Dictyostelium* cells by suppressing an Argonaute protein

**DOI:** 10.1186/s13100-015-0045-5

**Published:** 2015-09-03

**Authors:** Anika Schmith, Thomas Spaller, Friedemann Gaube, Åsa Fransson, Benjamin Boesler, Sandeep Ojha, Wolfgang Nellen, Christian Hammann, Fredrik Söderbom, Thomas Winckler

**Affiliations:** Department of Pharmaceutical Biology, Institute of Pharmacy, University of Jena, Semmelweisstrasse 10, 07743 Jena, Germany; Department of Molecular Biology, Biomedical Center, Swedish University of Agricultural Sciences, Uppsala, Sweden; Institute of Biology – Genetics, University of Kassel, Kassel, Germany; Ribogenetics@Biochemistry Lab, Department of Life Sciences and Chemistry, Molecular Life Sciences Research Center, Jacobs University Bremen, Bremen, Germany; Department of Cell and Molecular Biology, Biomedical Center, Uppsala University, Uppsala, Sweden; Present address: Aprea AB, Karolinska Institutet Science Park, Nobels väg 3, 17175 Solna, Sweden; Present address: Department of Biology, Brawijaya University, Jl. Veteran, Malang, East Java Indonesia

**Keywords:** *Dictyostelium*, Retrotransposition, siRNA, RNAi, Argonaute

## Abstract

**Background:**

In the compact and haploid genome of *Dictyostelium discoideum* control of transposon activity is of particular importance to maintain viability. The non-long terminal repeat retrotransposon TRE5-A amplifies continuously in *D. discoideum* cells even though it produces considerable amounts of minus-strand (antisense) RNA in the presence of an active RNA interference machinery. Removal of the host-encoded C-module-binding factor (CbfA) from *D. discoideum* cells resulted in a more than 90 % reduction of both plus- and minus-strand RNA of TRE5-A and a strong decrease of the retrotransposition activity of the cellular TRE5-A population. Transcriptome analysis revealed an approximately 230-fold overexpression of the gene coding for the Argonaute-like protein AgnC in a CbfA-depleted mutant.

**Results:**

The *D. discoideum* genome contains orthologs of RNA-dependent RNA polymerases, Dicer-like proteins, and Argonaute proteins that are supposed to represent RNA interference pathways. We analyzed available mutants in these genes for altered expression of TRE5-A. We found that the retrotransposon was overexpressed in mutants lacking the Argonaute proteins AgnC and AgnE. Because the *agnC* gene is barely expressed in wild-type cells, probably due to repression by CbfA, we employed a new method of promoter-swapping to overexpress *agnC* in a CbfA-independent manner. In these strains we established an in vivo retrotransposition assay that determines the retrotransposition frequency of the cellular TRE5-A population. We observed that both the TRE5-A steady-state RNA level and retrotransposition rate dropped to less than 10 % of wild-type in the *agnC* overexpressor strains.

**Conclusions:**

The data suggest that TRE5-A amplification is controlled by a distinct pathway of the *Dictyostelium* RNA interference machinery that does not require RNA-dependent RNA polymerases but involves AgnC. This control is at least partially overcome by the activity of CbfA, a factor derived from the retrotransposon’s host. This unusual regulation of mobile element activity most likely had a profound effect on genome evolution in *D. discoideum*.

**Electronic supplementary material:**

The online version of this article (doi:10.1186/s13100-015-0045-5) contains supplementary material, which is available to authorized users.

## Background

Transposable elements are found in virtually all organisms and play central roles in shaping their host’s genomes. The amplification of these genomic parasites is a constant threat to host fitness due to the intrinsic process of integration into the genomic DNA that can cause mutagenesis of genes and force illegitimate recombinations between distant transposon copies [1–4]. Eukaryotic cells have evolved several pathways of RNA interference (RNAi) to restrain the amplification of transposons at the posttranscriptional level [5–8]. In this process, long RNA duplexes (dsRNA), which may occur in cells as intermediates of transposon or RNA virus replication, are typically processed into 20–30 nucleotide double-stranded small interfering RNAs (siRNAs) by ribonuclease III-type enzymes such as Dicer. The siRNAs are loaded onto RNA-induced silencing complexes (RISCs), which are minimally composed of an Argonaute protein and a small RNA [9, 10]. Argonaute proteins are characterized by an RNA-binding PAZ (Piwi-Argonaut-Zwille) domain and a catalytic, ribonuclease H-like PIWI (P-element-induced wimpy testis) domain. Argonaute proteins bind siRNAs via their PAZ domains, unwind the siRNA duplex and use one of the single-stranded RNA molecules as guides to bind mRNAs in a sequence-specific manner [9]. If the guide RNA is fully complementary to the target RNA across the active site of the Argonaute protein, the enzyme is able to degrade the target RNA by a single endonucleolytic cut executed by the PIWI domain, a function termed slicing. If slicing is precluded by mismatches between the annealing guide RNA and cellular mRNA, translation is repressed and mRNA can be degraded by deadenylation and decapping.

The social amoeba *Dictyostelium discoideum* has a haploid genome in which nearly two thirds of DNA are protein-coding genes [11]. Despite the remarkable compactness of its genome, *D. discoideum* accommodates a large number of mobile elements that add up to approximately 10 % of the entire genomic DNA [12]. Most likely for the purpose of suppressing transposition, the organism has evolved a sophisticated RNAi machinery that includes, for example, three RNA-dependent RNA polymerases (RdRPs), two Dicer-like proteins, and five Argonaute-like proteins [13–17]. Intriguingly, the non-long terminal repeat retrotransposon TRE5-A has established a fairly high amplification rate in growing *D. discoideum* cells [18, 19] despite the constitutive production of minus-strand RNA from an element-internal promoter [20, 21]. Thus, how TRE5-A manipulates the cellular RNAi machinery to maintain its remarkable retrotransposition activity is of interest.

Clearly, *D. discoideum* cells could take advantage of TRE5-A’s minus-strand RNA production to downregulate TRE5-A plus-strand RNA, the substrate for retrotransposition, using an RNAi pathway. This strategy is actually realized in the silencing of the tyrosine recombinase retrotransposon DIRS-1 in *D. discoideum* cells [22]. To suppress TRE5-A amplification, promoter activity of the C-module, the distinguished minus-strand RNA promoter at the 3′ end of the TRE5-A element, could be positively regulated by a host-encoded transcription factor. This could elevate the level of TRE5-A-derived dsRNA, which could be processed into small RNAs that guide Argonaute proteins to degrade TRE5-A plus-strand RNA and prevent retrotransposition. Consistent with this idea, we previously isolated the C-module-binding factor (CbfA), a host-encoded DNA-binding protein that interacts with the C-module of TRE5-A in vitro [23–25].

The gene CbfA-coding could not be inactivated by conventional homologous recombination (knockout) and may be essential for the growth of *D. discoideum* cells. We constructed a knock-in mutant, JH.D, in which the *cbfA* gene was replaced by a *cbfA* variant containing an *amber* stop codon at amino acid position 455 [25]. The expression of an *amber* suppressor tRNA gene in *D. discoideum* cells allows read-through translation without causing an inherent phenotype [26]. Due to the low efficacy of this *amber* suppression, JH.D cells produce less than 5 % of full-length CbfA protein from the expressed *cbfA*(*amber*) mRNA [25].

JH.D cells have an aberrant developmental phenotype that can be fully rescued by ectopic expression of CbfA in the mutant [27]. Transcriptome analyses revealed that CbfA has general gene regulatory functions in *D. discoideum* cells [28], making this protein an attractive candidate as a host protein that could limit TRE5-A expression and retrotransposition by elevating TRE5-A-derived minus-strand RNA. Interestingly, we observed that both plus- and minus-strand RNA of TRE5-A were reduced concurrently in the CbfA mutant by more than 90 %, and this reduction of transcript levels was accompanied by a sharp drop in TRE5-A’s retrotransposition activity in vivo [21]. Remarkably, the promoter activity of neither the A-module (TRE5-A’s plus-strand RNA promoter) nor the C-module was altered in reporter gene assays in the CbfA mutant compared to wild-type cells [21]. Thus, we hypothesized that CbfA supports TRE5-A amplification indirectly by down-regulating one or several components of the cellular RNAi machinery. In support of this assumption, a previous transcriptome analysis revealed an approximately 230-fold and 3-fold overexpression of the genes encoding *D. discoideum* Argonaute-like proteins AgnC and AgnE, respectively, in the CbfA-depleted mutant [28].

Here, we found that TRE5-A expression was elevated in knockout strains of *agnC* and *agnE*, suggesting that CbfA may support the accumulation of TRE5-A transcripts by suppressing an RNAi pathway that involves these Argonaute proteins. To determine whether control of TRE5-A expression by AgnC and/or AgnE leads to a reduction in TRE5-A retrotransposition in vivo, we first developed a new gene activation (GA) strategy to construct strains that overexpress *agnC* in the absence of any residual plasmid sequences inserted in their genomes. We found that the accumulation of TRE5-A RNA was reduced in both *agnC*^*GA*^ and *agnE*^*GA*^ strains. Next, we employed the previously developed “TRE trap” retrotransposition assay [18, 19] to determine the retrotransposition activity of the cellular TRE5-A population in *agnC*^*GA*^ cells. The retrotransposition frequency of the cellular TRE5-A population was determined to be less than 10 % of the wild-type level, suggesting that TRE5-A amplification in *D. discoideum* cells is under surveillance of a distinct RNAi pathway that requires AgnC function and that this control of mobile element expansion is at least in part overcome by CbfA, a factor derived from the retrotransposon’s host cell.

## Results

### CbfA regulates the expression of the Argonaute-like protein AgnC

**Fig. 1:**
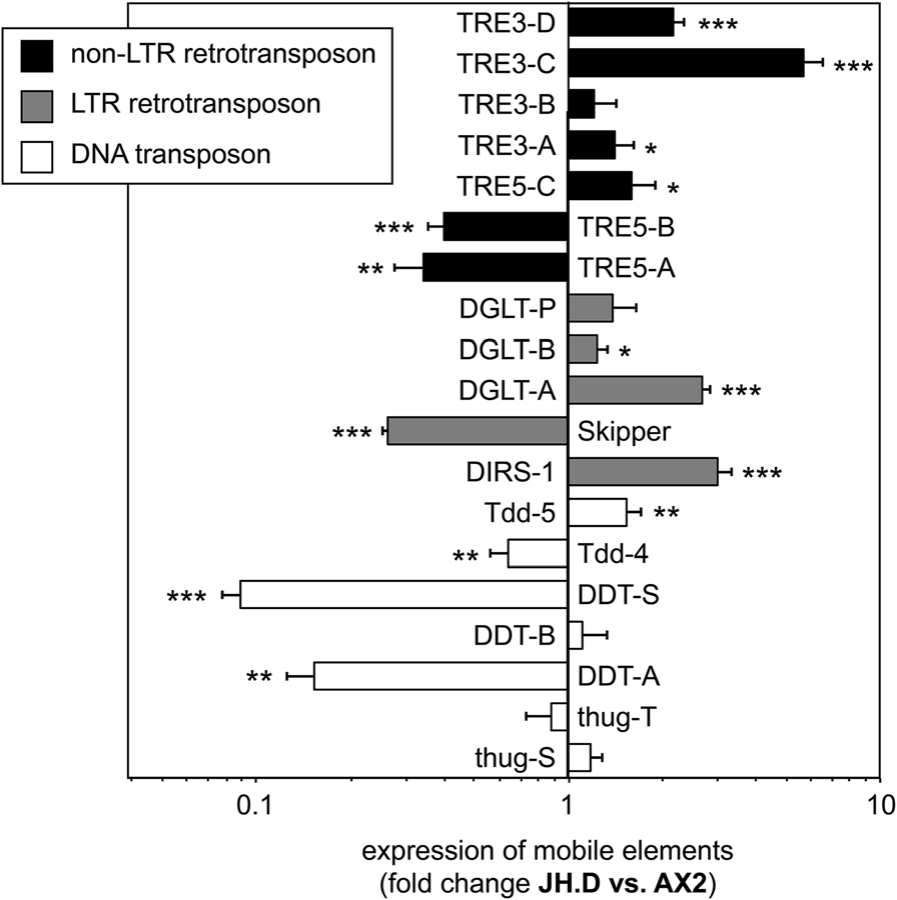
Expression of mobile elements in the CbfA underexpressing mutant JH.D. Expression data are derived from a previous RNA-seq analysis [28]. RPKM values (reads per kb of mRNA and standardized to 1 million reads) were obtained for the indicated mobile elements and calculated as ratio of JH.D versus AX2 and are shown as “fold change” of expression, meaning that values >1 represent overexpression of genes in JH.D. Values are means from three independent cultures ± SD. *p < 0.05, **p < 0.01, ***p < 0.001, relative to control AX2 cells (Student’s t-test). Black bars indicate non-LTR retrotransposons, grey bars indicate LTR retrotransposons (including the tyrosine recombinase retrotransposon DIRS-1), and white bars indicate putative DNA transposons

Even though the accumulation of TRE5-A RNA in *D. discoideum* cells strictly depends on CbfA and this factor binds to the C-module of TRE5-A in vitro, it does not regulate the C-module’s promoter activity in vivo [21]. A probable explanation for this paradox could be that CbfA exerts an indirect effect by regulating an RNAi pathway that is involved in the control of TRE5-A expression. In concordance with a rather indirect and probably broader function of CbfA in the control of mobile elements, including TRE5-A, the re-evaluation of previously obtained mRNA-seq data [28] suggested a considerable amount of deregulation of transposable elements in the CbfA-depleted mutant JH.D compared to the parental strain AX2 (Fig. 1). Typically the differential expression of mobile elements between AX2 and JH.D cells was highly variable among different cell cultures and could not be unequivocally verified by quantitative RT-PCR. Nevertheless, this observation strengthened the hypothesis that the regulation of steady-state levels of TRE5-A RNA may not be directly regulated by CbfA by binding to the C-module, but rather be the result of CbfA regulating an RNAi pathway involved in the regulation of TRE5-A.

**Fig. 2:**
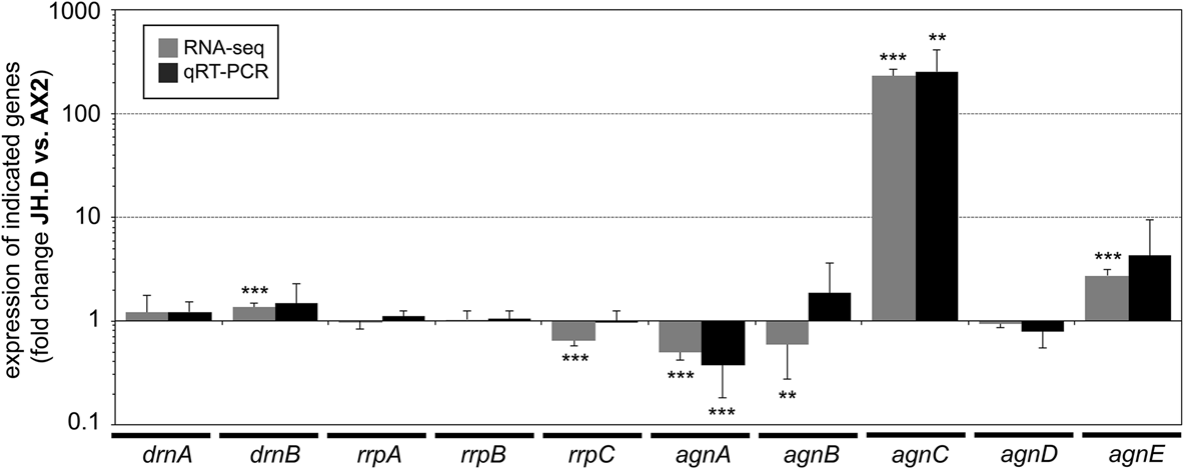
Expression of RNAi components in CbfA mutant JH.D cells. Expression of Dicer-like proteins (*drnA*, *drnB*), RdRPs (*rrpA-C*) and Argonaute genes (*agnA-E*) was analyzed by RNA-seq (gray bars, n = 3) in wild-type AX2 and CbfA-mutant JH.D cells from three independent cultures [28]. These three RNA samples, and RNA from three additional independent cultures, were analyzed by qRT-PCR (black bars, n = 6). Data are shown as fold change of expression with values >1 meaning that expression in the mutant cells was higher than in the control cells. Values are means ± SD from three and six independent cultures, respectively. **p < 0.01, ***p < 0.001, relative to wild-type AX2 cells (Student’s t-test)

Following this hypothesis we used previously obtained mRNA-seq data [28] to determine differential expression of putative RNAi components between the CbfA mutant JH.D and parent AX2 cells. The Argonaute genes *agnC* and *agnE* were 228-fold and a 2.7-fold, respectively, overexpressed in JH.D cells (Fig. 2). The genes coding for Argonaute proteins AgnA and AgnB and the RdRP RrpC were slightly underexpressed in the JH.D mutant cells, whereas expression of the genes coding for the RdRPs *rrpA* and *rrpB* were unaffected by CbfA depletion (Fig. 2). RNA-seq also revealed normal expression of the genes coding for the two Dicer-like proteins of *D. discoideum*, *drnA* and *drnB*, in the CbfA mutant (Fig. 2). To confirm the RNA-seq data, we determined the expression the Dicer genes, the three RdRPs, and the five Argonaute genes by qRT-PCR. For these measurements we combined three RNA samples used in the previous RNA-seq experiment with RNA preparations from three additional independent cultures. The data were consistent in all six biological replicates and are presented in Fig. 2. The strong overexpression of *agnC* in the CbfA mutant was confirmed (257-fold, p < 0.01, Student’s t-test). The weak overexpression of *agnE* seen in RNA-seq could not be verified by qRT-PCR at a statistically significant level (4.3-fold overexpression; p = 0.17), although the trend to *agnE* overexpression in JH.D was reproduced (Fig. 2). The weak but highly significant underexpression of *agnA* in the JH.D mutant observed by RNA-seq (2.1-fold; p < 0.001) was confirmed by qRT-PCR (3.6-fold; p < 0.01), whereas results for *agnB* were inconclusive (Fig. 2).

**Fig. 3:**
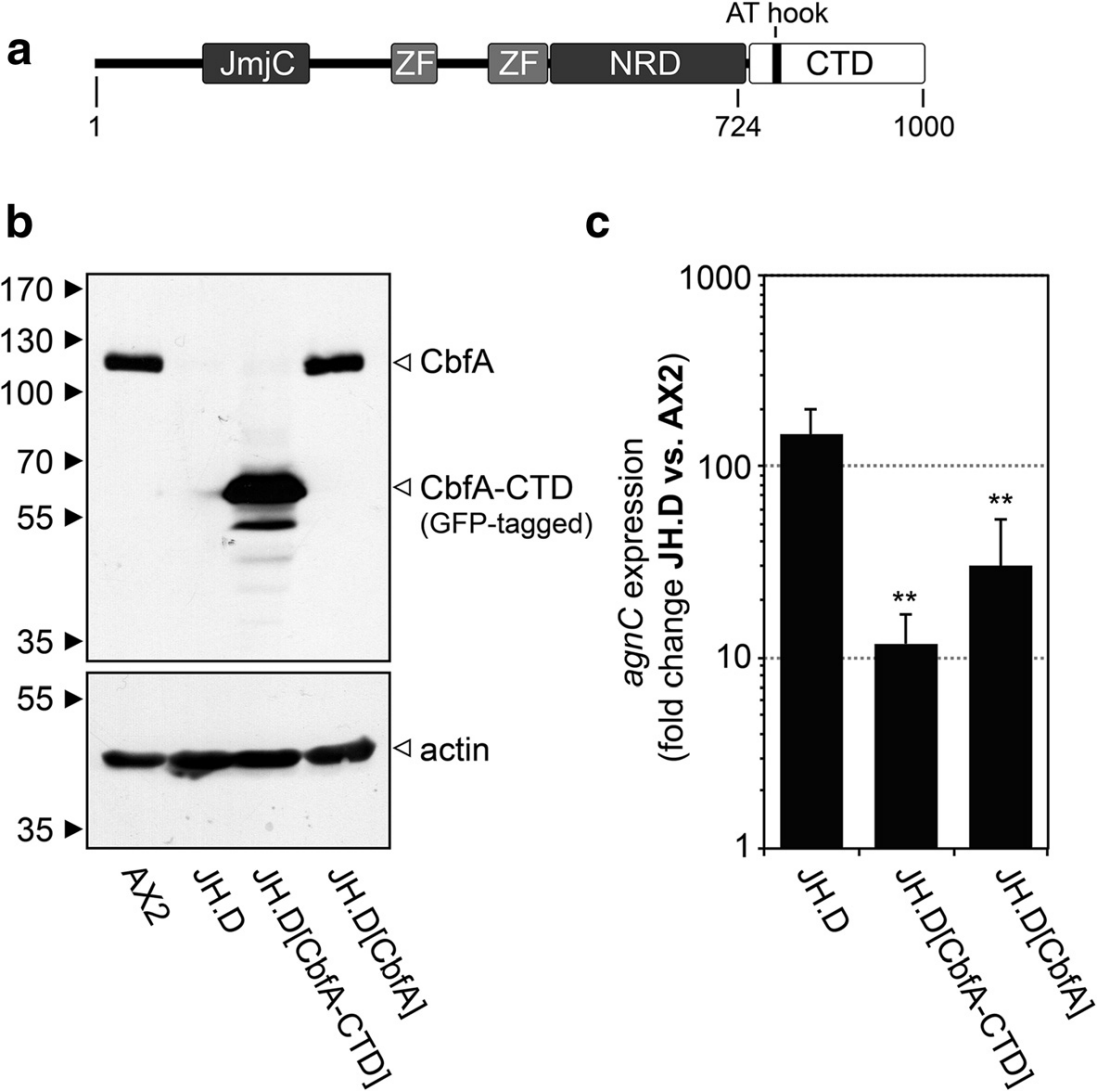
CbfA controls AgnC expression. **a** Scheme of the CbfA protein. The 1000-amino acid protein can be roughly divided into an amino-terminal part that may have chromatin-remodeling activity and a carboxy-terminal part that may facilitate DNA-binding [28, 39]. JmjC: carboxy-terminal jumonji domain", ZF: zinc finger-like motif; NRD: asparagine-rich domain; CTD: carboxy-terminal domain. The CbfA proteins expressed in mutant JH.D comprised either the full length protein (amino acids 2–1000) or the CbfA-CTD (amino acids 724–1000). **b** Expression of CbfA in JH.D cells. CbfA mutant JH.D cells were transformed with plasmids allowing for the expression of either untagged, full-length CbfA [21] or the GFP-tagged, carboxy-terminal domain of CbfA (CbfA-CTD) [28]. Shown is a western blot of whole-cell extracts prepared from the indicated strains. CbfA was visualized with the monoclonal antibody 7 F3 that detects CbfA-CTD. Numbers to the left indicate the sizes of the protein standards in kDa. **c** Complementation of the *agnC* overexpression phenotype in JH.D cells. Expression of *agnC* in the indicated strains was determined by qRT-PCR. Expression levels in JH.D cells and JH.D transformants were compared to AX2 wild-type cells and are expressed as “fold change” of expression, meaning that values >1 represent overexpression of genes in the JH.D strains and a value of 1 would indicate complete reversion of the overexpression in JH.D cells. Values are means from six independent cultures ± SD. **p < 0.01, relative to control AX2 cells (Student’s t-test)

CbfA can be divided into an amino-terminal part containing a “carboxy-terminal jumonji domain” and two zinc finger-like motifs, as well as a carboxy-terminal domain (CbfA-CTD) that contains a DNA-binding AT hook motif (Fig. 3a). We have previously determined that the CbfA-CTD is able to mediate most of CbfA’s gene-regulatory activity without requiring the rest of the CbfA protein [28] and it also completely restores TRE5-A expression in JH.D cells [21]. We wanted to evaluate whether the aberrant expression of *agnC* and *agnE* in JH.D cells was reversed by expression of CbfA in the mutant. To this end, we expressed either full-length CbfA or a GFP fusion to CbfA-CTD in JH.D cells using multicopy expression plasmids as shown in Fig. 3b. To confirm functional expression of CbfA or CbfA-CTD in JH.D, we first determined TRE5-A expression (Additional file 1: Figure S1). TRE5-A underexpression was rescued to wild-type level by full-length CbfA and was even “over-complemented” by CbfA-CTD, which was likely due to overexpression of this protein relative to normal CbfA amounts present in AX2 cells (compare Fig. 3b). The overexpression of *agnC* in JH.D was reduced by full-length CbfA by 80 % (p < 0.05) and by CbfA-CTD by 92 % (p < 0.05) (Fig. 3c). This result was similar to previous RNA-seq data [28], which revealed 97 % reduction (p < 0.001) of *agnC* overexpression in response to the presence of CbfA-CTD [28]. Taken together, the data indicate that *agnC* is a genuine CbfA-regulated gene that requires CbfA-CTD for proper expression. The data argued for a role of AgnC in CbfA-dependent TRE5-A regulation because the accumulation of TRE5-A transcripts is also regulated by CbfA-CTD [21].

Expression of full-length CbfA in JH.D cells had only a minor effect on the observed overexpression of *agnE* in JH.D cells. Likewise, expression of CbfA-CTD in JH.D cells did not affect *agnE* overexpression in the CbfA mutant (Additional file 1: Figure S1). The latter results were consistent with previous RNA-seq data, which did not indicate an effect of CbfA-CTD on *agnE* expression [28]. Therefore, we cannot definitely conclude from our data that *agnE* is a genuine CbfA-regulated gene.

### AgnC and AgnE downregulate TRE5-A expression

**Fig. 4:**
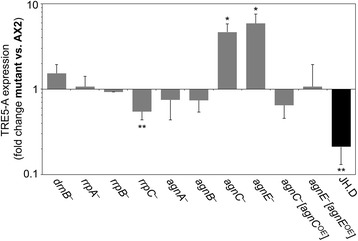
Expression of TRE5-A in knockout mutants of RNAi components. Expression of TRE5-A ORF1 was analyzed by qRT-PCR in the indicated knockout mutants of Argonaute genes and the Dicer-like protein DrnB. Phenotype reversion in *agnC* and *agnE* knockouts was accomplished using TAP-tagged *agnC* and *agnE* overexpressed in the respective mutants (*agnC* ^*−*^[*agnC* ^*OE*^] and *agnE* ^*−*^[ *agnE* ^*OE*^]). TRE5-A expression in JH.D cells is shown for comparison. Expression levels were compared to AX2 wild-type cells and are expressed as fold change of expression, meaning that values >1 represent overexpression of genes in the mutants. Data represent means from three independent cultures ± SD. *p < 0.05, **p < 0.01 relative to AX2 cells (Student’s t-test)

We performed qRT-PCR measurements to determine whether putative components of the *D. discoideum* RNAi machinery are involved in the silencing of TRE5-A expression. No significant changes of TRE5-A expression were determined when the genes coding for the Dicer-like protein DrnB or the RdRP proteins RrpA and RrpB were inactivated; however, we detected a mild but significant underexpression of TRE5-A in *rrpC* knockout cells (Fig. 4). Inactivation of *agnA* or *agnB* had no effect on TRE5-A expression (Fig. 4), whereas a 4.3- and 5.9-fold overexpression of TRE5-A was observed in knockout mutants of *agnC* and *agnE*, respectively (Fig. 4). Overexpression of TRE5-A in *agnC* and *agnE* knockout strains was completely reversed when AgnC or AgnE was expressed from a multicopy plasmid in the respective knockout mutant (Fig. 4), suggesting that both AgnC and AgnE contribute to TRE5-A regulation.

The role of AgnC and AgnE in the suppression of the TRE5-A population could be analyzed in more detail if the corresponding genes would be overexpressed in a wild-type background, i.e., in a strain with normal CbfA activity. This was an important consideration because previous data indicated that CbfA may have functions in TRE5-A retrotransposition beyond the regulation of TRE5-A RNA levels in supporting the integration process upstream of tRNA genes [21]. Usually, overexpression of proteins in wild-type cells is facilitated by transforming cells with expression plasmids. We assumed that this would be a suboptimal strategy for our experiments because transformants would contain insertions of multicopy plasmids at random genomic positions that could compromise the subsequent determination of retrotransposition activity of the TRE5-A population using the TRE trap assay (see below). With this consideration in mind, we decided to generate gene activation (GA) strains in which the endogenous promoter of either *agnC* or *agnE* was replaced by the strong *actin15* promoter (*act15P*) by homologous recombination. The advantage of this approach would be that the resulting overexpressor strains had stable genetic modifications at known genomic locations with an absence of remaining plasmid sequences, rather than random (multicopy) plasmid insertions, which have been shown to generate somewhat aberrant expression [29].

**Fig. 5:**
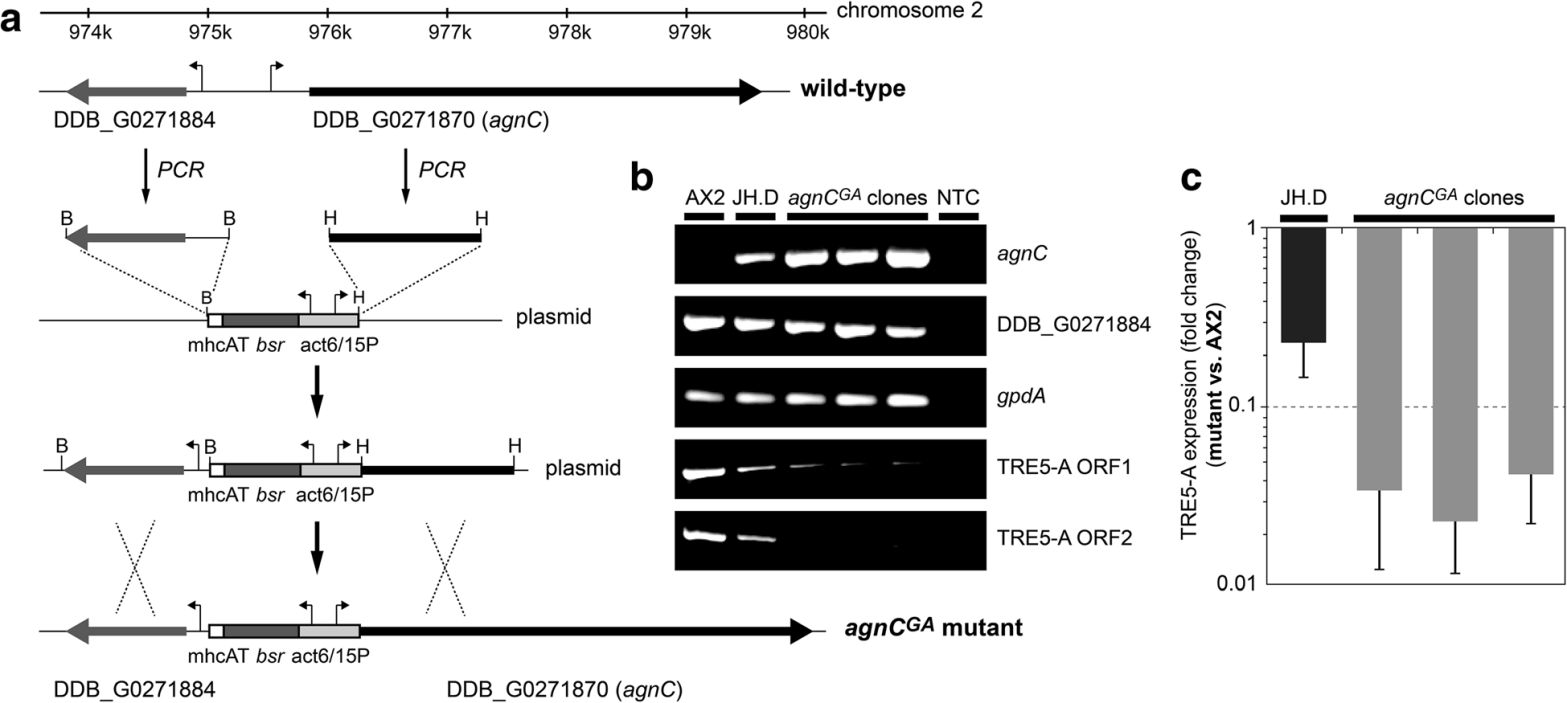
TRE5-A expression in *agnC* ^*GA*^ mutants. **a** Construction of *agnC* “gene activation” mutants. The *agnC* locus on chromosome 2 is indicated by nucleotide positions. The gene activation cassette consisted of a hybrid *actin6/actin15* promoter (arrows indicate transcription direction). The BamHI arm contained a 1200 bp DNA fragment covering the complete coding sequence of gene DDB_G0271884, including 273 bp of upstream sequence. The HindIII arm contained 1200 bp of *agnC* coding sequence, including the original translation start site. After double-recombination of the *agnC* ^*GA*^ vector with genomic DNA, the expression of *agnC* was driven by the *act15* promoter, whereas expression of the neighboring gene DDB_G0271884 was unaffected. **b** Semi-quantitative RT-PCR analysis of RNA from AX2, JH.D, and three independent *agnC* ^*GA*^ mutants demonstrating overexpression of *agnC*, normal expression of the neighboring gene DDB_G0271884 and *gpdA* (loading control), and silencing of TRE5-A (ORF1 and ORF2 sequences). NTC: no template control. **c** Quantitative RT-PCR of TRE5-A (ORF1) expression on RNA from JH.D and three *agnC* ^*GA*^ mutants. Expression levels were compared to AX2 cells and are expressed as fold change of expression, meaning that values <1 represent lower levels of TRE5-A in the mutants relative to wild-type AX2 cells. Data represent means from four independent cultures of the indicated strains ± SD

The *agnC* locus on chromosome 2 is indicated in Fig. 5a. The *agnC* gene shares its upstream sequence with gene DDB_G0271884, which is transcribed in the opposite direction. The gene activation construct consisted of a DNA fragment containing a fused *actin6*/*actin15* promoter element, which allowed for the expression of a blasticidin resistance gene under the control of *act6P* and the *agnC* gene under the control of *act15P* in opposite directions (Fig. 5a). A 1200 bp fragment of the *agnC* gene, including its authentic translation start, was inserted downstream of the *act15* promoter, whereas another 1200 bp fragment covering the complete coding sequence of gene DDB_G0271884, including 273 bp of upstream sequence, was cloned downstream of the blasticidin resistance cassette to generate a classical two-armed knockout plasmid. After double homologous recombination in the transformants, 749 bp of upstream *agnC* sequence were replaced by the *act15* promoter. PCR on genomic DNA from several independent *agnC*^*GA*^ mutants confirmed the promoter exchange and ensured that the organization at the locus was otherwise unaffected, particularly with respect to the upstream gene DDB_G0271884.

Semi-quantitative RT-PCR of *agnC*^*GA*^ mutants revealed strong overexpression of *agnC* in growing cells, whereas the transcript was barely detectable in wild-type cells (Fig. 5b). Whereas a 23-fold overexpression of *agnC* in JH.D cells relative to wild-type AX2 cells was determined by qRT-PCR, AX2[*agnC*^*GA*^] strains contains between 995- to 2016-fold excess *agnC* mRNA compared to AX2 cells. Yet the *agnC*^*GA*^ mutants had no obvious phenotype during growth and multicellular development. Expression of the *agnC*-upstream gene DDB_G0271884 was not affected by the homologous recombination yielding the *agnC*^*GA*^ strains (Fig. 5b). Semi-quantitative RT-PCR revealed that the TRE5-A steady-state transcript level in the *agnC*^*GA*^ mutants dropped sharply and was even more lower than in JH.D cells (Fig. 5b). This was confirmed by qRT-PCR, which suggested 24- to 45-fold lower expression of the TRE5-A ORF1 transcript in *agnC*^*GA*^ mutants than in wild-type cells, compared to a 4-fold decrease seen in JH.D cells (Fig. 5c). The data suggested that AgnC is directly involved in suppressing TRE5-A transcripts in *D. discoideum* cells.

Strains overexpressing the *agnE* gene in the AX2 background were constructed by employing the gene activation strategy as outlined in Additional file 1: Figure S2. Apparently overexpression of AgnE in the recovered *agnE*^*GA*^ strains resulted in an average of 2.3-fold underexpression of TRE5-A in four strains tested, thus confirming that AgnE may be involved in TRE5-A suppression.

### AgnC is a suppressor of TRE5-A retrotransposition

**Fig. 6:**
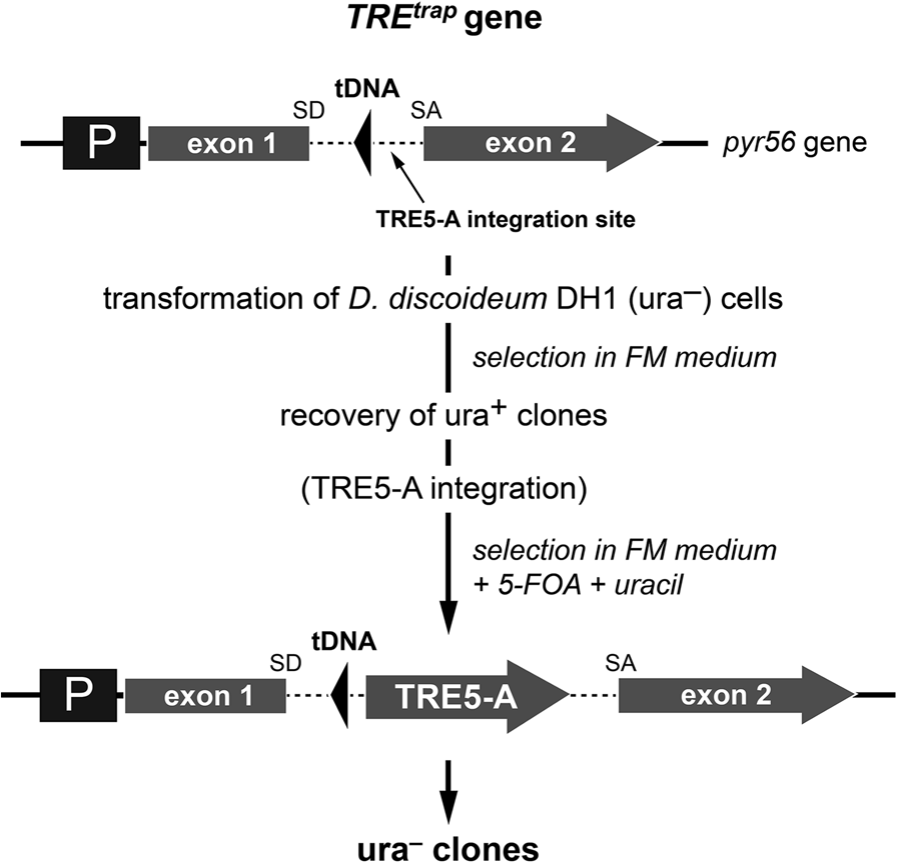
Outline of the TRE trap retrotransposition assay. The *TRE* ^*trap*^ gene is a modified version of the *pyr56* gene, which codes for the *D. discoideum* UMP synthase. The *pyr56* gene contains an intron (dashed line; SD: splice donor site, SA: splice acceptor site) into shich a tRNA gene is inserted as bait for the integration of TRE5-A. The *TRE* ^*trap*^ gene is transformed into the ura^−^ strain DH1 that has a complete deletion of the *pyr56* gene. The *TRE* ^*trap*^ gene converts transformants to ura^+^ because the intron is functionally spliced. If an element of the endogenous TRE5-A population targets the tRNA gene in the *TRE* ^*trap*^ gene for integration, the *TRE* ^*trap*^ gene is disrupted even if the integration actually occurs in the intronic sequence. The resulting ura^−^ cells gain resistance to the drug 5-fluoroorotic acid (5-FOA) and grow out clonally if uracil is added to the medium [18]

It seemed plausible that silencing of TRE5-A expression would diminish retrotransposition of the TRE5-A population in the *agnC* and *agnE* overexpressor strains. To directly measure the retrotransposition activity of the cellular TRE5-A population, we set up a previously described “TRE trap” in vivo retrotransposition assay [18, 19] in *agnC*^*GA*^ cells. The TRE trap is based on a modified *pyr56* gene (*TRE*^*trap*^) that codes for UMP synthase (Fig. 6). The *TRE*^*trap*^ gene contains an intron into which a *Val*^*UAC*^ tRNA gene was inserted as target for TRE5-A integrations. After transformation of the *TRE*^*trap*^ gene into ura^−^ cells, the transformants present a ura^+^ phenotype due to the expression of functional UMP synthase from the *TRE*^*trap*^ gene (with the intron including the tRNA gene being spliced out); however, the cells are prone to mutations in the *TRE*^*trap*^ gene by integration of cellular TRE5-A elements upstream of the embedded tRNA gene. Cells affected by TRE5-A integration into the *TRE*^*trap*^ gene can no longer splice out the intron and are converted to the ura^−^ phenotype; they can be recovered after clonal growth in medium complemented with 5-fluoroorotic acid (5-FOA) and uracil. In previous studies, approximately 100 insertions into the TRE trap were analyzed for integration by mobile elements [18, 19]. The data revealed that ~1 % of recovered ura^−^ clones had spontaneous loss-of-function mutations of the *TRE*^*trap*^ gene, whereas ~99 % of the clones carried a TRE5-A element. No insertions of other members of the tRNA gene-specific TRE retrotransposon family were detected in this assay, suggesting that they amplify at a very low rate. Thus, the number of clones obtained in the TRE trap assay is an estimate of the TRE5-A retrotransposition activity in *D. discoideum* cells [18, 19].

**Fig. 7:**
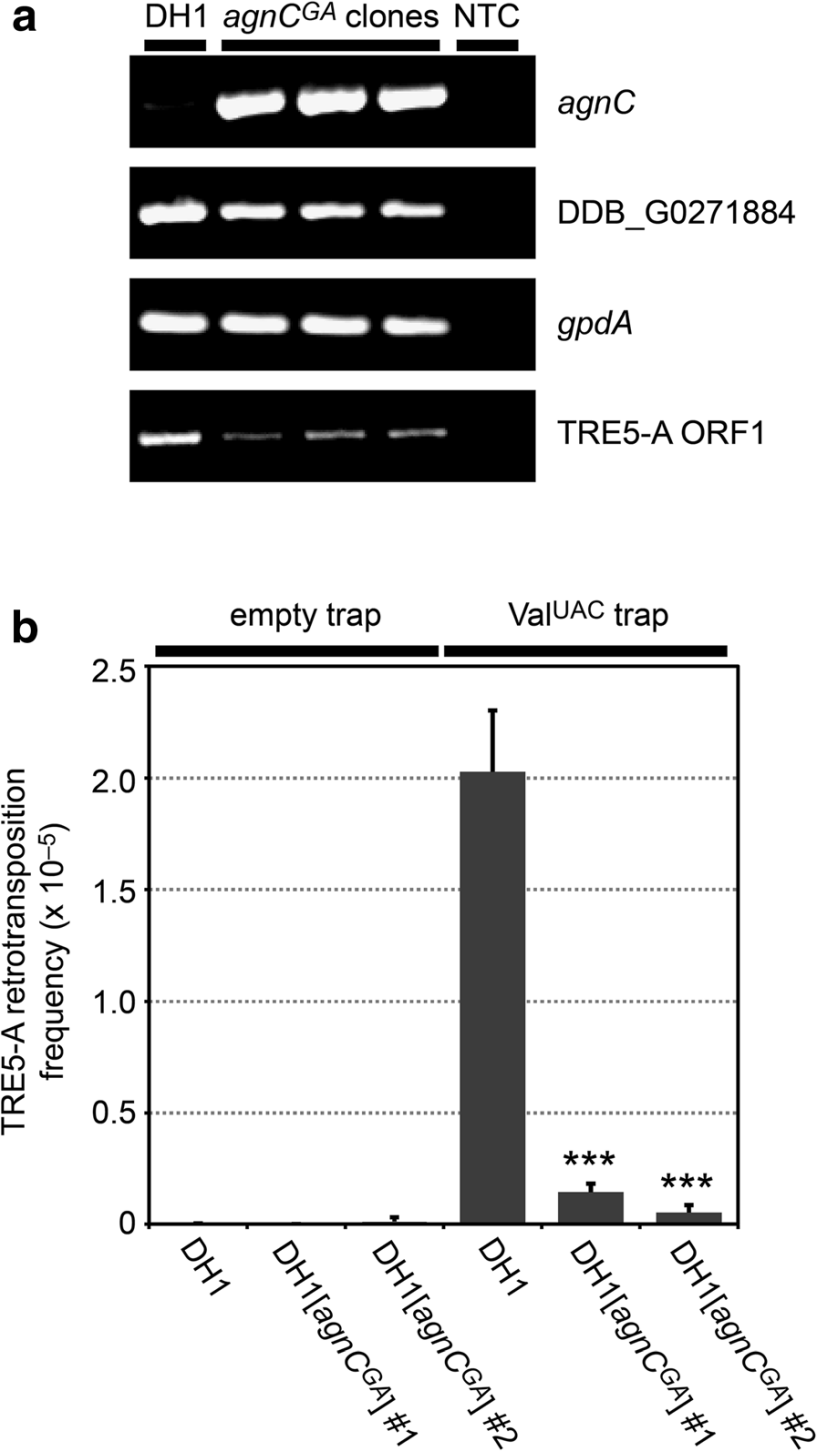
Retrotransposition of the TRE5-A population in *agnC* overexpressing strains. **a** Semi-quantitative RT-PCR analysis of RNA from DH1 and three independent DH1[*agnC* ^*GA*^] mutants. Note that *agnC* was overexpressed comapred to DH1. Expression of the neighboring gene DDB_G0271884 (compare Figure 5) was not affected, whereas expression of TRE5-A ORF1 was reduced in the *agnC* ^*GA*^ mutants. Expression of the house-keeping gene *gpdA* is shown as control. NTC: no template control. **b** DH1 and DH1[*agnC* ^*GA*^] cells were transformed with either the empty *TRE* ^*trap*^ gene (i.e., no tRNA gene inserted in the intron) or the *TRE* ^*trap*^ gene containing a *Val* ^*UAC*^ tRNA gene. In this TRE trap assay, one clone of DH1 transformants and two independent clones of DH1[*agnC* ^*GA*^] cells (clones #1 and #2) were analyzed for TRE5-A retrotransposition. For each strain, five plates containing 10^7^ cells each were prepared. Cells were cultured in FM medium containing 5-FOA and uracil until clones appeared. Clones were counted and results are presented as retrotransposition frequency ± SD. ***p < 0.001 (Student’s t-test). This assay was reproduced once with similar results

In the experiments described above, AX2 was the parent strain for the generation of *agnC*^*GA*^ and *agnE*^*GA*^ mutants because we wanted to be able to directly compare them to JH.D cells, which were also derived from AX2. Because no suitable uracil-auxotrophic AX2 mutant was available, we reproduced *agnC*^*GA*^ mutants in the ura^−^ strain DH1, which is an AX3 derivative [30]. As shown in Fig. 7a, overexpression of *agnC* in the recovered DH1[*agnC*^*GA*^] strains was comparable to AX2[*agnC*^*GA*^] cells. Likewise, TRE5-A ORF1 expression was suppressed in DH1[*agnC*^*GA*^] strains, albeit at somewhat lower efficacy as in AX2[*agnC*^*GA*^] cells. qRT-PCR revealed that TRE5-A expression in the particular DH1[*agnC*^*GA*^] strain that we subsequently used to determine TRE5-A retrotransposition was 7.7-fold lower than in the parental DH1 strain. This DH1[*agnC*^*GA*^] strain was transformed with plasmids carrying either the empty TRE trap (i.e., no tRNA gene inserted in the trap) or the *TRE*^*trap*^ gene, which contained a *Val*^*UAC*^ tRNA gene as bait for TRE5-A integrations. Five plates, each containing 10^7^ cells, were cultured in minimal medium supplemented with 5-FOA and uracil until clones appeared. As the positive control, the TRE5-A retrotransposition frequency in DH1[*TRE*^*trap*^] cells was determined at 2.03 × 10^−5^, whereas it was <0.01 × 10^−5^ in DH1[*TRE*^*trap*^] cells in which the *Val*^*UAC*^ tRNA gene was omitted as the negative control. In two independently recovered DH1[*agnC*^*GA*^/*TRE*^*trap*^] strains, TRE5-A retrotransposition activity was determined at 0.14 × 10^−5^ and 0.05 × 10^−5^, representing a more than 90 % drop retrotransposition in the *agnC* overexpressing cells compared to control cells (p < 0.001, Student’s t-test) (Fig. 7b). These data indicate that AgnC controls the amplification of TRE5-A elements in *D. discoideum* cells by limiting the accumulation of retrotransposon-derived RNA.

Although we were able to establish *agnE*^*GA*^ strains in the DH1 background, we could not recover viable cells after transformation of the *TRE*^*trap*^ gene into these cells. Therefore, we were unable to determine the retrotransposition activity of the TRE5-A population under these conditions. Thus, it remains elusive at this point whether the moderate downregulation of TRE5-A transcripts in *agnE*^*GA*^ strains correlates with an appreciable suppression of the retrotransposition activity of the TRE5-A population.

## Discussion

### AgnC and AgnE act in RNAi pathways to suppress TRE5-A retrotransposition

Because the TRE5-A element produces both plus- and minus-strand RNA, we assumed that the retrotransposition frequency of the TRE5-A population may be under surveillance by the cellular RNAi machinery. In this study, we provide evidence to support this assumption. Genetic inactivation of either AgnC or AgnE resulted in overexpression of TRE5-A, suggesting that both proteins have functions in TRE5-A regulation in which the loss of one cannot be compensated for by expression of the other. The *D. discoideum* Argonaute proteins are a part of the PIWI subfamily of Argonaute proteins. They all have divergent amino-terminal domains, but possess conserved PAZ and PIWI domains including an intact DEDH catalytic tetrad and probably possess slicer activity. One model to explain TRE5-A silencing is the generation of PIWI-interacting RNAs (piRNAs) by AgnC and AgnE in a ping-pong piRNA replication mechanism typical for PIWI proteins [31]. This model is intuitive given that piRNAs are often generated from long single-stranded RNA precursors produced from transposable elements, such as the minus strand RNA of TRE5-A. However, piRNA are usually 23–30 nt long [31, 32], which contradicts results from a previous deep sequencing of small RNA libraries which revealed the formation of ~21 nt siRNAs from TRE5-A elements at a very low level (0.05 % of total small RNAs) in growing *D. discoideum* cells [15].

The silencing of the retrotransposon DIRS-1 is a model to study RNAi pathways in *D. discoideum* [14, 15, 17, 22]. Previous deep sequencing of small RNAs revealed high levels of DIRS-1-derived ~21 nt siRNAs [15] that add up to 20 % of all small RNAs detected in *D. discoideum* cells [17]. The difference in the amount of ~21 nt siRNAs derived from DIRS-1 and TRE5-A may be interpreted as the less efficient dsRNA formation from TRE5-A RNA compared to DIRS-1 RNA. DIRS-1 silencing is enhanced by the RdRP RrpC that synthesizes new DIRS-1 dsRNA that can be diced into secondary siRNAs [16]. This amplification step may be missing in the RNAi pathway that controls TRE5-A expression, because TRE5-A transcripts were not stabilized in RdRP-deficient mutants. The Dicer homolog DrnB, which is mainly required for miRNA formation in *D. discoideum* [15], is apparently not involved in the regulation of DIRS-1 RNA levels [17] and it seems to be also dispensable in the process of TRE5-A regulation (Fig. 4). Thus, the RNAi pathways that regulate DIRS-1 and TRE5-A may overlap at the stage of primary siRNA formation, presumably involving the Dicer homolog DrnA, but use different RISCs that contain either AgnA for DIRS-1 silencing [22] or AgnC/AgnE for TRE5-A suppression.

### CbfA abrogates TRE5-A suppression by repressing AgnC

The C-module at the 3′ end of the TRE5-A element has promoter activity that is responsible for the production of minus-strand RNA by the element [20]. CbfA was originally identified as a “C-module-binding factor” because it binds to the C-module of TRE5-A in vitro [24], but it does not regulate the C-module promoter activity in vivo [21]. Considering that CbfA regulates more than 1000 genes of the *D. discoideum* genome, the present data suggest that the observations of in vitro binding of CbfA to the C-module and in vivo regulation of steady-state levels of TRE5-A transcripts by CbfA are purely coincidental. Together with the data obtained in this study, we propose instead that the accumulation of TRE5-A transcripts in *D. discoideum* cells is indirect and a result of the CbfA-mediated suppression of a posttranscriptional pathway involving AgnC. This assumption is supported by the observation that both the plus- and minus-strand RNA of TRE5-A simultaneously vanish upon removal of CbfA from cells, but reappear when CbfA is re-introduced into CbfA-underexpressing cells [21]. In a previous mRNA-seq experiment comparing gene expression in JH.D with wild-type cells [28] we detected underexpression of putative DNA transposons such as DDT-A and DDT-S, the long-terminal repeat (LTR) retrotransposon Skipper, and the non-LTR retroransposon TRE5-B (see Fig. 1). RNA-seq also predicted overexpression of some mobile elements in the absence of CbfA such as the tyrosine recombinase retrotransposon DIRS-1, the LTR retrotransposon DGLT-A, and the non-LTR retrotransposons TRE3-C and TRE3-D. However, differential expression of the mentioned mobile elements in JH.D cells could not be unequivocally confirmed by qRT-PCR. This was obviously due to high biological variation between independent cultures that was never observed when analyzing the expression of coding genes (i.e., Fig. 2) and the reason for this phenomenon remains elusive. At least the overexpression of DIRS-1 in JH.D cells could be explained by the weak, but reproducible overexpression of the genes *rrpC* and *agnA* (Fig. 2), which were both shown to be involved in the downregulation of DIRS-1 [22]. Thus, DIRS-1 and TRE5-A may be suppressed by different RNAi pathways and are affected indirectly by CbfA’s broad-ranging gene-regulatory activity. Unfortunately, a high variability of mobile element expression among biological replicates was also observed when analyzing either *agnC* knockout or *agnC* overexpressor cells. Therefore, we are unable at this point to predict whether other mobile elements are regulated by the same AgnC-involving RNAi pathway that controls TRE5-A retrotransposition.

TRE5-A belongs to a family comprising seven tRNA gene-targeting retrotransposons in *D. discoideum* cells. The TRE5-A and TRE5-B elements are closely related and share a common ancestor, but only TRE5-A was amplified to a high copy number rather late in the evolution of this species [12]. It is puzzling that TRE5-A amplification in *D. discoideum* was apparently accelerated after the acquisition of the C-module (i.e., an antisense promoter) and after the split from its common ancestor with the TRE5-B element that lacks a C-module. Intuitively, the incorporation of an antisense promoter into a mobile element should make it vulnerable to silencing by RNAi-related mechanisms and thus prevent its amplification. Because the C-module was most likely acquired by 3′-transduction, a process not uncommon in this class of retrotransposons [33], the question of what advantage the element may have gained by incorporating an antisense promoter at its 3′ end remains. Did *D. discoideum* cells gain a selective advantage from TRE5-A expansion? The release of TRE5-A from RNAi surveillance by a regulated process involving a host-encoded factor such as CbfA may have evolved because it could be used for cellular purposes such as enhancing genome flexibility. Alternatively, TRE5-A release from suppression by RNAi may have been incidentally caused by adaptation to evolutionary pressure forcing alterations in AgnC-mediated posttranscriptional regulation that are unrelated to transposon suppression. It is unknown under which conditions the repression of *agnC* by CbfA would be released or for which functions AgnC would be required; at least, it seems to be unrelated to the multicellular development of *D. discoideum* because *agnC* is barely upregulated during development [34] and neither *agnC* knockouts nor *agnC* overexpressor display a developmental phenotype. Repression of AgnC by CbfA may provide an efficient way to respond to changes in particular environmental conditions that require specialized functions of this Argonaute protein. Even if this mode of gene regulation by CbfA would come at the cost of TRE5-A amplification, it is reasonably tolerable because TRE5-A’s targeted integration to regions upstream of tRNA genes would largely prevent insertion mutagenesis of the genome.

Whereas RNAi may have been developed to restrict mobile element expansion in *D. discoideum* as in other eukaryotes, as exemplified by DIRS-1 silencing, our study shows an intriguing example of a transposable element that is under surveillance by the cellular RNAi machinery, but the control of which can be overcome by suppression of a distinct RNAi pathway by a host factor.

## Conclusions

The social amoeba *D. discoideum* has a compact and haploid genome that requires tight control of mobile element activity to maintain genome stability. The non-long terminal repeat retrotransposon TRE5-A actively amplifies in the genome of *D. discoideum* even though the element should be vulnerable to posttranscriptional silencing due to the production of antisense RNA from an element-internal promoter. The host-encoded factor CbfA has global gene-regulatory functions in *D. discoideum* that include the suppression of the Argonaute-like proteins AgnC and AgnE. Whereas TRE5-A transcipts were found to accumulate in mutants lacking AgnC or AgnE, expression and retrotransposition of the element vanished in AgnC and AgnE overexpressing cells. These observations suggest that TRE5-A amplification is under surveillance by an RNAi pathway that involves AgnC and AgnE and that this control is at least partially overcome by the activity of CbfA. This unusual regulation of mobile element activity by a host factor most likely had a profound effect on genome evolution in *D. discoideum*.

## Methods

### Strains and plasmids

The CbfA-depleted mutant JH.D and the plasmids used for the expression of full-length CbfA and the GFP-tagged carboxy-terminal domain of CbfA (CbfA-CTD) have been previously described [21, 25]. *D. discoideum* strains harboring knockouts of RdRP genes *rrpA*, *rrpB*, and *rrpC* were described by Wiegand & Hammann [16]. Knockout strains of *agnA* and *agnB* were described elsewhere [22]. The *drnB*^*−*^ strain was described in Avesson et al., 2012 [35]. Knockout mutants of *agnC* and *agnE* as well as plasmids allowing for the expression of TAP-tagged AgnC and AgnE will be described in a separate publication (F.S. et al., manuscript in preparation).

### Construction of gene activation mutants

The *D. discoideum* expression vector pDM326 [36] contains a blasticidin resistance cassette driven by the *act6* promoter and an upstream *act15* promoter in opposite direction for the expression of transgenes. A DNA fragment containing both the blasticidin cassette and the *act15* promoter was isolated from pDM326 by digestion with BamHI and BglII. The DNA fragment was inserted into the BamHI site of pGEM7Zf(−) (Promega), such that the former BglII site was placed next to the HindIII site of the pGEM vector to generate pGEM-GA. To generate the *agnC*^*GA*^ vector, the “BamHI arm” covering the entire coding sequence of the gene DDB_G0271884, which shares its upstream region with *agnC*, was amplified including 273 bp of residual upstream sequence and inserted into the BamHI site of pGEM-GA. The “HindIII arm” was generated by amplification of nucleotides 1–1166 of the *agnC* gene including its authentic translation start codon. The pGEM-agnC-GA plasmid was linearized and transformed into *D. discoideum* AX2 or DH1 cells and transformants were selected in HL5 medium (Formedium, Hunstanton, UK) containing 6 μg/ml blasticidin (Life Technologies, Carlsbad, USA) [37]. From such clones genomic DNA was isolated and screened by PCR for insertion of the GA cassette at the targeted locus using one primer specific for the blasticidin resistance gene and a second primer that hybridized outside of the DNA sequences covered by the HindIII arm. RT-PCR was used to confirm that the expression of the *agnC*-upstream gene DDB_G0271884 was not affected by insertion of the GA cassette.

### Reverse transcription-PCR

Total RNA was prepared from frozen cell pellets and RT-PCR was done as described previously [21]. In quantitative RT-PCR gene expression levels were standardized to the gene coding for catalase (*catA*). The following qRT-PCR primers were used: rrpA-01, GAACGTCAAGAACTTGGTAAATTGTATC; rrpA-02, TAACCTACAGTTTGTAAC CGAATGTTTAC; rrpB-01, GAACGTCAAGAACTTGGTAAAATGTATAA; rrpB-02, GTGGATAACCTTTAGTTTTTAACCAAAC; rrpC-01, GGTGTTTATAGTAAAAAAGAATCATTC; rrpC-02, CAACTATCCAAGAATTTATGAACATTTAC; agnA-01, GCCGAAACTCCTTCTTCTTGGGGTAC; agnA-02, GTTCATCCAATAAGACATGGTAATGAG; agnB-01, GTGATGGTGTTGGTGATGGTATGTTAG; agnB-02, CTTGGTAATCCTGATCAAGGTGTTGTTG; agnC-03, GTGCACTTTTATGAGAGTATTGGCATAC; agnC-04, GTACATGATAATGAGTTGGATTTGTAG; agnD-01, CATCATATTAATAGTCGTTTACCAGAG; agnD-02, GTACCAATCCACCCAATGGTACAATGG; agnE-03, GAGCATAATTACAAGGAGCAGGTGTTC; agnE-04, CAGTGCTAACCATTGTACCATTGGGTG; catA-01, GTTTCGCTGCTCGTCAACCATACAATC; catA-02, GCACGAACTTGAATTTCTTTGATGGTG; gpdA-01, GGTTGTCCCAATTGGTATTAATGG; gpdA-02, CCGTGGGTTGAATCATATTTGAAC; TRE5-A ORF1 Rep-108, GTCATAAACATCAATCCGAACCAGAC; TRE5-A ORF1 Rep-109, GTTAGATTGTCTAGTTCAATGATAGTGTC; TRE5-A ORF2 Rep-75, GACTGTTCAGTGGATAATAACC; TRE5-A ORF2 Rep-176, CTCGAGTTAAAGGAAGATTGCTCTTGAATC; DDB_G0271884-01, GAGTTGGCCAAATTAGTTAAGCAATTG; DDB_G0271884-02, CCTTGTTCAACCCAAGAGAAAATTTCTG.

### TRE trap retrotransposition assay

The TRE trap is an in vivo retrotransposition assay that measures the activity of the cellular TRE5-A population. It was essentially performed as described previously [21]. The TRE trap consists of the complete *pyr56* gene modified to contain a functional intron into which a *Val*^*UAC*^ bait tRNA gene was inserted. This gene is referred to as the *TRE*^*trap*^ gene. After transformation into *D. discoideum* DH1 cells, ura^+^ cells harboring chromosomal integrations of the *TRE*^*trap*^ gene were recovered by cultivation in FM medium without supplements. After integration of a TRE5-A element into the trap, the *TRE*^*trap*^ gene is disrupted and no functional UMP synthase is expressed. Thus, affected cells were converted to the ura^−^ phenotype and gained resistance to the drug 5-fluoroorotic acid (5-FOA). In a typical retrotransposition assay, 5 plates each containing 10^7^ cells were prepared, and cells were cultured in FM medium containing 150 μg/ml 5-FOA and 20 μg/ml uracil. Clones that arose were counted, and the data presented are the means from 5 plates ± SD.

### Western blots

*D. discoideum* cells were washed in phosphate buffer and stored as frozen pellets of 2 × 10^7^ cells at −80 °C. SDS/polyacrylamide gel electrophoresis of whole-cell extract proteins and western blotting were done as described [38]. We used monoclonal antibody 7 F3 to detect CbfA and a polyclonal antiserum to detect actin8 [38].

### Additional data files

The following additional data are available with the online version of this paper. Additional file 1: Figure S1 shows the functional complementation of strain JH.D with CbfA or its carboxy-terminal domain with respect to TRE5-A and *agnE* expression. Additional file 1: Figure S2 illustrates the construction of *agnE*^*GA*^ mutants and shows the expression of TRE5-A in these mutants.

## Additional file

Additional file 1: Figure S1. Functional complementation of strain JH.D with CbfA or its isolated carboxy-terminal domain. RNA levels of retrotransposon TRE5-A (A) and *agnE* (B) were determined by qRT-PCR in JH.D cells, JH.D cells expressing full-length CbfA, and JH.D cells expressing CbfA-CTD. Expression levels in JH.D cells and JH.D transformants were compared to AX2 wild-type cells and are expressed as “fold change” of expression, meaning that values >1 represent overexpression of genes in the JH.D strains and a value of 1 would indicate complete reversion of the overexpression in JH.D cells. Values are means from six independent cultures ± SD. **p < 0.01, relative to control AX2 cells (Student’s t-test). Note that TRE5-A is actually overexpressed in the JH.D[CbfA-CTD] transformant, which is an effect of overexpression of CbfA-CTD (see Fig. 2, main text). **Figure S2.** TRE5-A expression in *agnE* ^*GA*^ mutants. (A) Construction of *agnE* “gene activation” mutants. The *agnE* locus on chromosome 5 is indicated by nucleotide positions. The gene activation cassette consisted of a hybrid *actin6/actin15* promoter (arrows indicate transcription direction). The BamHI arm contained a 1070 bp DNA fragment covering part of the coding sequence of gene DDB_G0289385. The HindIII arm contained 1080 bp of *agnE* coding sequence, including the original translation start site. After double-recombination of the *agnE* ^*GA*^ vector with genomic DNA, the expression of *agnE* was driven by the *act15* promoter, whereas expression of the neighboring gene DDB_G0289385 was unaffected. (B) Semi-quantitative RT-PCR analysis of RNA from AX2, JH.D, and three independent *agnE* ^*GA*^ mutants demonstrating overexpression of *agnE*, normal expression of the neighboring gene DDB_G0289385 and *gpdA* (loading control), and silencing of TRE5-A (ORF1 and ORF2 sequences). NTC: no template control. (C) Quantitative RT-PCR of TRE5-A (ORF1) expression on RNA from JH.D and four *agnE* ^*GA*^ mutants. Expression levels were compared to AX2 cells and are expressed as fold change of expression, meaning that values <1 represent lower levels of TRE5-A in the mutants relative to wild-type AX2 cells. Data represent means from four independent cultures of the indicated strains ± SD. (PDF 1033 kb)

## Abbreviations

CbfA: C-module-binding factor
CTD: carboxy-terminal domain (of CbfA)
GA: gene activation (by promoter swapping)
LTR: long terminal repeat
qRT-PCR: quantitative reverse-transcription PCR
RdRP: RNA-dependent RNA polymerase
RISC: RNA-induced silencing complex
RNAi: RNA interference
siRNA: small interfering RNA
TAP: tandem affinity purification
TRE5-A: tRNA gene-targeted retroelement 5-A
