# Supplementary material for: A host factor supports retrotransposition of the TRE5-A population in Dictyostelium cells by suppressing an Argonaute protein
Source: Mob DNA. 2015 Sep 3;6:14. doi: 10.1186/s13100-015-0045-5 (PMC4559204; doi:10.1186/s13100-015-0045-5)
Supplement: Additional file 1: Figure S1. — Functional complementation of strain JH.D with CbfA or its isolated carboxy-terminal domain. RNA levels of retrotransposon TRE5-A (A) and agnE (B) were determined by qRT-PCR in JH.D cells, JH.D cells expressing full-length CbfA, and JH.D cells expressing CbfA-CTD. Expression levels in JH.D cells and JH.D transformants were compared to AX2 wild-type cells and are expressed as “fold change” of expression, meaning that values >1 represent overexpression of genes in the JH.D strains and a value of 1 would indicate complete reversion of the overexpression in JH.D cells. Values are means from six independent cultures ± SD. **p < 0.01, relative to control AX2 cells (Student’s t-test). Note that TRE5-A is actually overexpressed in the JH.D[CbfA-CTD] transformant, which is an effect of overexpression of CbfA-CTD (see Fig. 2, main text). Figure S2. TRE5-A expression in agnE GA mutants. (A) Construction of agnE “gene activation” mutants. The agnE locus on chromosome 5 is indicated by nucleotide positions. The gene activation cassette consisted of a hybrid actin6/actin15 promoter (arrows indicate transcription direction). The BamHI arm contained a 1070 bp DNA fragment covering part of the coding sequence of gene DDB_G0289385. The HindIII arm contained 1080 bp of agnE coding sequence, including the original translation start site. After double-recombination of the agnE GA vector with genomic DNA, the expression of agnE was driven by the act15 promoter, whereas expression of the neighboring gene DDB_G0289385 was unaffected. (B) Semi-quantitative RT-PCR analysis of RNA from AX2, JH.D, and three independent agnE GA mutants demonstrating overexpression of agnE, normal expression of the neighboring gene DDB_G0289385 and gpdA (loading control), and silencing of TRE5-A (ORF1 and ORF2 sequences). NTC: no template control. (C) Quantitative RT-PCR of TRE5-A (ORF1) expression on RNA from JH.D and four agnE GA mutants. Expression levels were compared to AX2 cells and are expressed as fold c [file 13100_2015_45_MOESM1_ESM.pdf]

Additional File 1: Figure S1: Functional complementation of strain JH.D with CbfA or its isolated carboxy-terminal domain.

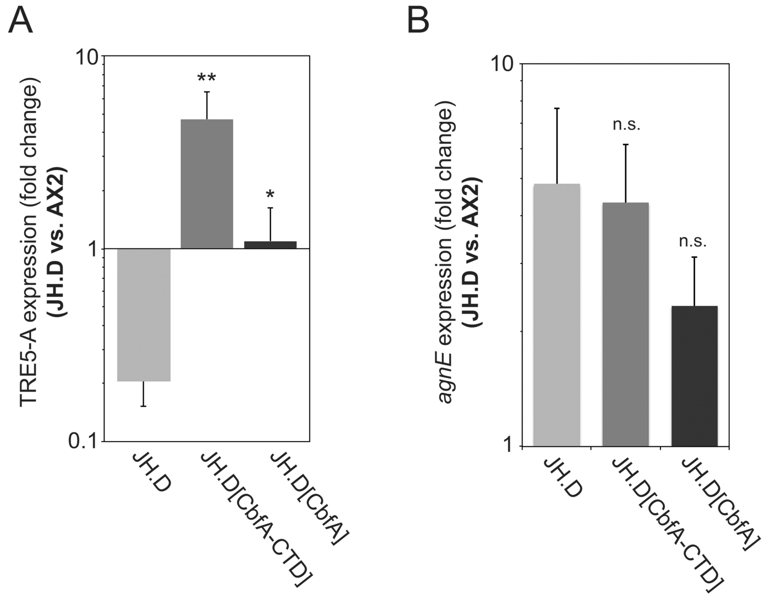

RNA levels of retrotransposon TRE5-A (A) and *agnE* (B) were determined by qRT-PCR in JH.D cells, JH.D cells expressing full-length CbfA, and JH.D cells expressing CbfA-CTD. Expression levels in JH.D cells and JH.D transformants were compared to AX2 wild-type cells and are expressed as "fold change" of expression, meaning that values >1 represent overexpression of genes in the JH.D strains and a value of 1 would indicate complete reversion of the overexpression in JH.D cells. Values are means from six independent cultures ± SD. **p<0.01, relative to control AX2 cells (Student's t-test). Note that TRE5-A is actually overexpressed in the JH.D[CbfA-CTD] transformant, which is an effect of overexpression of CbfA-CTD (see Figure 2, main text).

Additional File 1: Figure S2: TRE5-A expression in *agnE^GA* mutants.

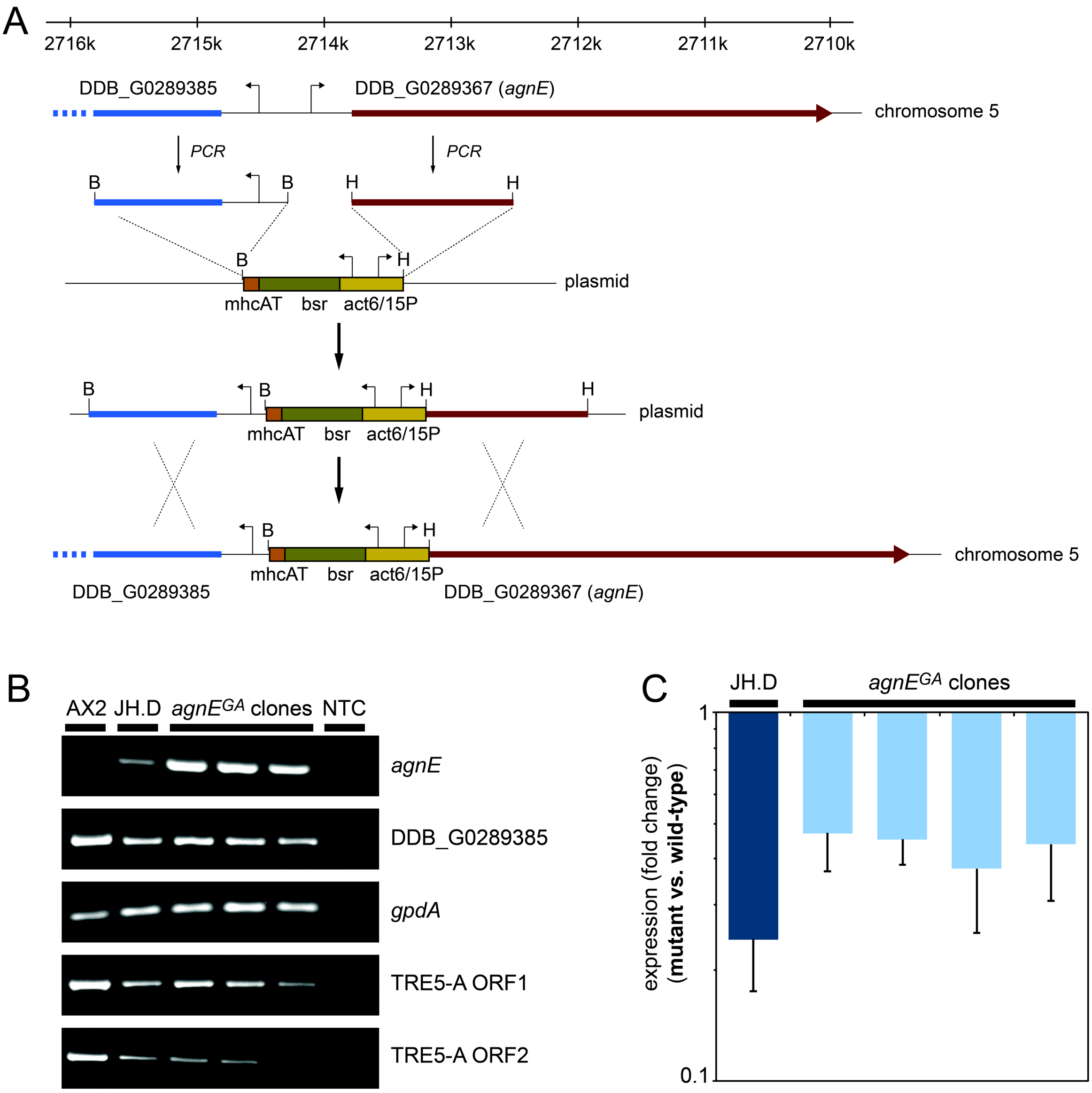

(A) Construction of *agnE* "gene activation" mutants. The *agnE* locus on chromosome 5 is indicated by nucleotide positions. The gene activation cassette consisted of a hybrid *actin6/actin15* promoter (arrows indicate transcription direction). The BamHI arm contained a 1070 bp DNA fragment covering part of the coding sequence of gene DDB_G0289385. The HindIII arm contained 1080 bp of *agnE* coding sequence, including the original translation start site. After double-recombination of the *agnE^GA* vector with genomic DNA, the expression of *agnE* was driven by the *act15* promoter, whereas expression of the neighboring gene DDB_G0289385 was unaffected. (B) Semi-quantitative RT-PCR analysis of RNA from AX2, JH.D, and three independent *agnE^GA* mutants demonstrating overexpression of *agnE*, normal expression of the neighboring gene DDB_G0289385 and *gpdA* (loading control), and silencing of TRE5-A (ORF1 and ORF2 sequences). NTC: no template control. (C) Quantitative RT-PCR of TRE5-A (ORF1) expression on RNA from JH.D and four *agnE^GA* mutants. Expression levels were compared to AX2 cells and are expressed as fold change of expression, meaning that values <1 represent lower levels of TRE5-A in the mutants relative to wild-type AX2 cells. Data represent means from four independent cultures of the indicated strains ± SD.
